# Editorial: Chemical contaminants in natural environments and human health implications

**DOI:** 10.3389/ftox.2025.1528372

**Published:** 2025-03-11

**Authors:** Aina Olubukola Adeogun, Azubuike Victor Chukwuka, Beatrice Olutoyin Opeolu, Oju Ibor, Ebenezer Olatunde Farombi

**Affiliations:** ^1^ Department of Zoology , University of Ibadan, Ibadan, Oyo, Nigeria; ^2^ National Environmental Standards and Regulations Enforcement Agency (NESREA), Abuja, Nigeria; ^3^ BEE Solutions and Consultancy Services, Cape Town, South Africa; ^4^ Department of Zoology and Environmental Biology, University of Calabar, Calabar, Cross River, Nigeria; ^5^ Department of Biochemistry, College of Medicine, University of Ibadan, Ibadan, Nigeria

**Keywords:** chemical contaminants, environmental pollution, human health implications, regulatory frameworks, risk mitigation

Natural environments are invaluable resources necessary for ecological and human health and chemical contaminants are increasingly recognized as significant threats to public health and environmental integrity. The growing body of research highlights their harmful effects and other human existential threats such as climate change currently add to the burden of pollution effects on the environment. These underscore the urgent need for effective regulatory frameworks and public health initiatives. Manuscripts that focus on topical \issues of chemical contaminants were selected for inclusion in this special edition of Frontiers in Toxicology.

A comprehensive analysis by Zhang et al. examined Bisphenol A (BPA) exposure among the Chinese population from 2004 to 2019, revealing alarmingly high BPA levels, particularly among males and children. The geographical variations in exposure were linked to waste disposal practices, emphasizing the critical need for improved waste management strategies to mitigate BPA exposure and associated health risks. This study not only highlights the health risks posed by BPA but also provides timely information for policymakers to prioritise sustainable waste management practices.

The threat posed by agricultural pollutants is similarly concerning, as illustrated in the study by Abarikwu et al., which focused on atrazine, a widely used herbicide. Authors demonstrated that atrazine induce d cytotoxicity, oxidative stress, and apoptosis, with detrimental effects on reproductive health, including decreased sperm count. Notably, authors observed that antioxidants like vitamin E could alleviate these toxic effects, suggesting potential strategies for risk mitigation. This finding raises the possibility of integrating antioxidant supplements into agricultural practices to counteract pesticide toxicity, thereby protecting both agricultural workers and consumers.

Expanding on the narrative of environmental pollution, Alarape et al. assessed glyphosate residues in African catfish across various markets in Ibadan, Nigeria, and reported residue concentrations exceeding acceptable limits. This finding underscores the urgent need for stricter pesticide regulations in aquaculture to safeguard consumer health and aquatic ecosystems. Coupled with this, Akinnusotu et al. identified polycyclic aromatic hydrocarbons (PAHs) in sediment and fish from River Owan, Edo State. Although their study indicated minimal cancer risks associated with these contaminants, the presence of pyrogenic PAHs highlights the need for ongoing monitoring to prevent long-term ecological damage and protect aquatic biodiversity.

In the context of water quality, Mhlongo et al. investigated the occurrence of phenolic compounds in potable and treated waters in the Western Cape of South Africa. Although detected at levels below regulatory thresholds, health risk assessment suggested potential non-carcinogenic effects and slight mutagenicity. This underscores the need for vigilance in water quality management, with recommendations for routine monitoring and stricter enforcement of regulations to ensure safe drinking water for all communities.

The impact of environmental chemical exposures on children’s mental health is a pressing concern, as highlighted in the narrative review by James and Oshaughnessy. A comprehensive review of 29 studies revealed significant associations between exposure to heavy metals and endocrine disruptors and adverse mental health outcomes in children. This emphasises the critical need for further research into the cumulative effects of these chemical agents during sensitive developmental stages. Policymakers must prioritize educational campaigns and preventive measures to reduce children’s exposure to these harmful substances, ensuring a healthier future generation.

Akangbe et al. investigated endocrine-disrupting chemicals in fish from Lagos and Epe lagoons, providing significant gonadal alterations and hormonal imbalances insights, with some fish exhibiting intersex characteristics. This research illustrates the profound impacts of environmental contaminants on aquatic life and highlights the necessity for targeted management strategies to protect these ecosystems. Collaborative efforts among researchers, regulators, and local communities can foster sustainable practices to mitigate these risks in aquatic biota and ensure human health.

Occupational exposure to chemical contaminants was examined by Barros et al. in a study of wildland firefighters, revealing elevated levels of PAH metabolites and increased blood pressure among participants, with higher risks for smokers. This finding emphasises the urgent need for protective measures in high-risk professions and the development of guidelines to minimise occupational exposure to hazardous chemicals.

Lead exposure remains a critical concern, as demonstrated by He et al., whose study measured blood lead levels in residents of Jiangxi Province. Findings suggest that higher lead levels correlated with adverse haematological and biochemical indices, particularly among older adults. This underscores the widespread health risks associated with lead exposure in vulnerable populations and highlights the necessity for comprehensive screening programs and public health initiatives aimed at reducing lead exposure in communities. In a related study, Zhang et al. explored the link between heavy metal exposure and persistent infections, revealing that exposure to metals such as arsenic, cadmium, and lead increased infection risks primarily through immunosuppression. This finding further illustrates the intricate relationship between environmental contaminants and human health, necessitating a multi-faceted approach to address these interconnected issues.

In this study, Tian et al. examined the relationship between perfluoroalkyl and polyfluoroalkyl substances (PFASs) and glucose metabolism, indicating that higher PFAS exposure correlated with increased fasting plasma glucose levels and decreased insulin levels. This association highlights the potential metabolic consequences of chemical exposure, necessitating further investigation into the long-term health effects of PFAS on human health.

Collectively, these studies present a concerning picture of the pervasive threats posed by chemical contaminants to both human health and environmental integrity and underscore the urgent need for robust regulatory frameworks, public health initiatives, and ongoing research to mitigate exposure risks and protect vulnerable populations.

Priority actions for next steps in environmental and human health protection entails adopting proactive approaches that include:

- Implementing stricter regulations on the use of harmful chemicals in agriculture and industry.
- Promoting public awareness campaigns to educate communities about the dangers of chemical exposure and preventive measures.
- Enhancing monitoring and enforcement of water quality standards to protect public health.
- Encouraging interdisciplinary collaborations among researchers, policymakers, and communities to develop and implement effective solutions.

By prioritising these actions, we can address the pressing challenges posed by chemical contaminants and safeguard human and ecosystems health for sustainable exploitation of their abundant resources.

